# Auditory Accommodation to Poorly Matched Non-Individual Spectral Localization Cues Through Active Learning

**DOI:** 10.1038/s41598-018-37873-0

**Published:** 2019-01-31

**Authors:** Peter Stitt, Lorenzo Picinali, Brian F. G. Katz

**Affiliations:** 1 Audio Acoustics Group, LIMSI, CNRS, Université Paris-Saclay, Orsay, France; 2 Dyson School of Design Engineering, Imperial College London, London, UK; 3 Sorbonne Université, CNRS, Institut Jean Le Rond d’Alembert, F-75005 Paris, France

## Abstract

This study examines the effect of adaptation to non-ideal auditory localization cues represented by the Head-Related Transfer Function (HRTF) and the retention of training for up to three months after the last session. Continuing from a previous study on rapid non-individual HRTF learning, subjects using non-individual HRTFs were tested alongside control subjects using their own measured HRTFs. Perceptually worst-rated non-individual HRTFs were chosen to represent the worst-case scenario in practice and to allow for maximum potential for improvement. The methodology consisted of a training game and a localization test to evaluate performance carried out over 10 sessions. Sessions 1–4 occurred at 1 week intervals, performed by all subjects. During initial sessions, subjects showed improvement in localization performance for polar error. Following this, half of the subjects stopped the training game element, continuing with only the localization task. The group that continued to train showed improvement, with 3 of 8 subjects achieving group mean polar errors comparable to the control group. The majority of the group that stopped the training game retained their performance attained at the end of session 4. In general, adaptation was found to be quite subject dependent, highlighting the limits of HRTF adaptation in the case of poor HRTF matches. No identifier to predict learning ability was observed.

## Introduction

An important aspect of human audition is spatial perception of sound and, more specifically, sound source localization. Humans decode auditory spatial information from the signals reaching both ears using binaural difference cues and spectral processing^1^. Binaural difference cues contain information about the lateralization of the perceived source, and include Interaural Time Difference (ITD) and Interaural Level Difference (ILD). Spectral cues are used for estimating source elevation and front/back discrimination. Distance perception relies on these and additional cues, which have been the subject of studies, often integrating visual^2,3^ and dynamic cues^3^ as well as environmental factors.

Both binaural and spectral cues are encoded in the Head Related Transfer Function (HRTF), which is composed of a set of filter pairs (one for each ear) for various positions around the head. The HRTF characterizes the acoustic signal transformations to the two ears, including head shadowing and diffraction, torso and pinnae reflections, from each specific source direction. It therefore includes features which are dependent on the morphology of each listener, such as the size of their head and shape of their pinnae.

The HRTF for a given source position can be used to filter a single channel monophonic signal for playback over stereo headphones, thereby simulating the presence of a virtual sound source in the surrounding space. This process is known as binaural spatialization or, more precisely, binaural synthesis. Difficulties in the perceptual processing of spatial cues arise when sound sources are spatialized using a non-individualized HRTF, *i.e*. using a set of filters measured from an individual other than the listener. This can result in inaccurate source localization, *e.g*. confusion between front/back and up/down, and in problems related to the externalization of virtual sound sources^4^.

An individual’s HRTF can be obtained using an anechoic chamber and specialized measurement procedures and equipment. This, in addition to the time required for the measurement, makes it difficult to provide each potential listener with their own HRTF for a personalized experience. A number of approaches have been developed to provide personalized HRTFs, including numerical simulation^5–7^, subjective selection^8,9^, tuning of spectral cues, and scaling based on morphological criteria^10,11^. Each of these methods presents challenges in terms of the computational and technical complexity required to obtain an individual HRTF. For this reason, single or “generic” HRTFs are often used in many binaural systems, meaning that a significant number of users are likely to be listening to inappropriate spatial cues.

An alternative and viable approach for obtaining acceptable spatialization performances using non-individual HRTFs is listener adaptation. Humans have been shown to be able to adapt to changes in their HRTF, as shown by a number of previous studies^12–17^. A review of studies evaluating human adaptation to modified or non-individual spectral cues can be found in^18^.

Previous studies on adaptation to new spectral cues have tended to use broadband noise stimuli. Encouragingly, a recent study^19^ showed that training to broadband stimuli in an anechoic environment generalizes to different stimuli (speech), untrained positions, and to echoic environments. This was determined using pinnae insert molds, which the subjects wore during waking hours for 10 days, and training sessions taking advantage of sensory-motor feedback information. This further demonstrates the validity of using broadband stimuli during training to altered spectral cues or non-individual HRTFs.

Using a similar approach to earlier studies^13,15^, recently Trapeau *et al*.^20^ used pinnae insert molds which subjects wore for 6 days, tracking the adaptation through regular localization performance tests. Improvements were found over the course of the 6 days for both azimuth and elevation performances. Testing again 1 week, and 1 month, after training showed that 70%, and then 60%, of the adaptation was retained. It should be noted that the reported study was unable to differentiate between a decrease in performance due to loss of adaptation to modified spectral cues or to lack of exposure to the localization test procedure.

Considering the duration of the adaptation effect, Zahorik *et al*.^21^ found that improvement in terms of front-back reversals remained for a period of 4 months after training. Shorter-lasting improvements were found by Mendonça *et al*.^22^, although the study only tested elevations in the front-upper quadrant and their methodology excluded the possibility of reporting front-back confusions. For subjects in the main test group, who repeated the localization test at each session, improvement was retained for 1 month. However, the control condition groups, which included subjects who were only tested once after the training (a day, week, or month later), showed a continuous decrease of the improvement with time. However, it must to be underlined that the tested population comprised only three subjects in each group in the control condition, making it hard to draw significant conclusions.

Parseihian & Katz^23^ showed that humans have the ability to adapt to non-individual HRTFs cues through a series of short training sessions. This study employed an active auditory-kinesthetic process as the method for HRTF adaptation, which was found to provide a decrease in localization error using just 3 training sessions of 12 min each over the course of several days. Three HRTF groups were tested: the perceptually best-rated, the worst-rated, and the individually measured control ones. As expected, the group using their individual HRTFs was found to obtain the best results on all measures of error. The groups with non-individual HRTFs showed improvement in terms of localization, increasing with repetitions of the training game. The group using their best-rated HRTFs improved more quickly than those using their worst-rated. It was hypothesized that further training sessions would be required to improve the performance of the group using their worst-rated HRTF. After three training sessions, the group using their best-rated HRTFs approached localization performance levels of the individual HRTF control group.

## Summary and Aim of the Present Study

Previous studies have investigated adaptation to non-individual HRTFs or modified spectral cues^13,20–23^, sometimes with elements of investigation into the retention of this adaptation.

To further investigate this, this work takes a step forward from one previous study^23^, and aims at examining both the perceptual adaptation to non-individual HRTFs and the retention of the improvement over a long-term period. Aiming at reproducing the worst-case scenario in any Virtual Auditory Environment (VAE) in which the HRTF cannot be personalized, the current study evaluates only the HRTFs perceptually *worst-rated* by subjects. This allows also for the maximum potential improvement over the whole experiment. The study’s protocol was developed to examine the following four points raised from earlier studies.

### Point 1 – Training interval

In contrast to the earlier study of Parseihian & Katz^23^, which used an auditory-kinesthetic training method with a protocol of 3 training sessions on successive days, the current study examines if an increase in the delay between sessions has an impact on the success of auditory accommodation. To examine this, sessions 1–4 took place at intervals of 1 week, followed by 6 sessions at intervals of 2 weeks, for a total of 10 sessions. During the first series of 4 sessions, all subjects performed the training game and localization test. For the second series, sessions 5–10 (every 2 weeks), subjects with non-individualized HRTFs were split into two groups: one continued to perform both the localization test and the training game (group **W10**), while the other continued only with the localization test (group **W4**). Intervals of 1–2 weeks were chosen as being significantly greater than the previous study employing training on consecutive days, while still maintaining a reasonable (not excessive) duration of engagement of all participants. The test protocol structure is summarized in Table 1.

**Table 1:** Summary of the different group definitions and whether or not the Training game and Localization test were carried out during the various test sessions by the test subjects and control group.

ID	HRTF	Sessions 1–4		Sessions 5–10	
		Train.	Loc.	Train.	Loc.
W4	worst-rated	✓	✓	✕	✓
W10	worst-rated	✓	✓	✓	✓
C10	individual	✓	✓	✓	✓

### Point 2 – Training repetitions

The results of previous studies have pointed to a potential plateau^23^ or oscillatory^13^ effect in spectral accommodation, and poor/limited success in the worst-case scenario. Extending the number of training sessions from 3 to 10 for group **W10** allows for longer accommodation time in this HRTF condition compared to the previous study using the same protocol.

### Point 3 – Accommodation perennity

In order to examine the quality of retention of any achieved adaptation, continued localization performance tests are used in the current study to track localization ability over the 3 months after training has stopped for group **W4**. These repeated evaluations allow for investigation of whether previously observed^20,22^ degradations of the improvements in localization performance after training are due to spectral unlearning (*i.e*. accommodation is a short term effect), rather than to lack of exposure to the test protocol.

### Point 4 – Individual variations

Previous studies have shown clear performance differences between individuals, with some exhibiting degraded or oscillatory performance over repeated sessions^13^. The performance of group **W10**, carrying out the training for the entire 10 sessions, is essential to determine the extent to which perceptually worst-rated HRTFs can be learned given an increased opportunity to adapt. Can the same degree of accommodation be achieved by all subjects in this worst-case scenario? Time-intervals between sessions and the duration of training sessions were kept as similar as possible across participants to provide consistent test conditions.

The results of both groups are compared to those of the control group (**C10**) which trained using their own individual HRTFs, in order to separate procedural learning (where participants merely become familiar with the test protocol and the potential spatial distortions introduced by the system) from perceptual learning (where participants’ interpretation of the HRTF’s spectral cues changes).

To summarize, the previous points are transformed into the corresponding 4 hypothesis tested in the current study:

**H1** Adaptation to the perceptually worst-rated HRTF set can be achieved with intervals of 1–2 weeks between training sessions. (point 1)

**H2** Continued training sessions allow for consistent improvement in localization for the perceptually worst-rated HRTF set. (points 1 & 2)

**H3** Improvement in localization accuracy from training is retained well beyond the training period, supporting the results of ^21,22^. (point 3)

**H4** Adaptation to the perceptually worst-rated HRTF is possible for any unimpaired individual. (point 4)

## Methods

### Subjects

A total of 20 adult subjects (6 women, age between 21 and 46 years, mean 31 years) served as paid volunteers (150€ for the series of 10 sessions beginning in February 2016); no subjects self-reported any hearing deficit. Of these subjects, 4 used their previously measured individual HRTFs, while the remaining 16 were assigned non-individual HRTFs. Subjects were naive to the purpose of the experiment and the sets of spatial positions selected for the experiment. Of these, 4 were familiar with VAEs, with 2 of these 4 belonging to the control group. Informed consent was obtained from all participants.

All procedures were carried out in accordance with the relevant guidelines and regulations. The test protocol was developed in accordance with the Declaration of Helsinki (DoH) concerning research protocols involving human subjects that do not aim to advance biological or biomedical knowledge. According to the Comité d’évaluation éthique de l’Inserm (CEEI/IRB) and French regulations, this study did not require submission and approval by the committee of protection of persons (CPP).

### Non-Individual HRTF Selection

The non-individual HRTFs employed in the text were comprised of a set of 7 HRTFs selected from 46 HRTFs of the public database LISTEN^24^ to be perceptually orthogonal as per^9^, and further validated by^25^. The selection method of the set of 7 HRTFs ensured that each HRTF was satisfactory for at least some of the population. This ensured that no HRTF was included which was considered globally bad (*i.e*. lacking in reliable localization cues).

**Figure 1:**
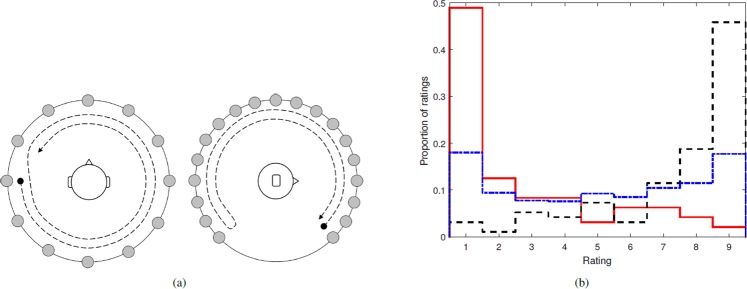
Non-individual HRTF selection stimuli and results. (**a**) Descriptions for HRTF quality ratings: horizontal (left) and median (right) plane trajectories indicating the start/stop position and trajectory direction (•→). (**b**) Cumulative distribution of HRTF ratings. The distribution for ratings across all HRTFs (−⋅−). Distribution of collected individual ratings of the identified (**—**) *worst*- and (**– –**) *best*-matched HRTFs across all subjects. Distribution is normalized by the total number of ratings performed ((3× median plane + 3× horizontal plane)×16 subjects).

A VAE subjective quality judgment test was used in order to rate the different HRTFs for each subject, using a discrete 9-point scale with extremes being “best” and “worst” by evaluating the rendering of two predefined source trajectories. The two trajectories were described to the subjects, allowing them to form an internal reference against which they could rate the stimuli (see Fig. 1a). The first trajectory was a circle in the horizontal plane with source positions at 30° spacing. The trajectory began directly left and followed two complete rotations around the subject. The second trajectory followed an arc in the median plane (azimuth 0°) from elevation −45° in front to −45° at the rear with positions at 15° spacing. The trajectory commenced at −45° in front, proceeded to the top and to −45° in the rear, and then returned along the same path to the front. The stimuli source was a repeated noise burst, 0.23 s in duration, and shaped with a Hann function window (see^25^ for additional details). In order to eliminate ITD cues from the HRTF rating task, the ITD of each of the 7 HRTFs was estimated (using the *maxIACC* method^26^), removed, and replaced by the mean ITD across the 7 HRTFs for the corresponding position. As such, ITDs were common across the rated HRTFs.

Subjects to be assigned non-individual HRTFs judged the 7 HRTF sets for each trajectory. Following the results of ^27^, which examined the reliability and repeatability of HRTF judgments by naive and experienced subjects, this rating task was performed 3 times, leading to a total of 6 ratings per subject counting the 2 trajectories. An overall judgment rating was taken as the mean of the two trajectory judgments across repetitions. The highest and lowest rated HRTF for each subject were then used as that subject’s perceptually *best* and *worst*-match HRTF respectively. The distributions of ratings across subjects and repetitions for all HRTFs, as well as the resulting *best*- and *worst*-rated HRTFs, are reported in Fig. 1b. While the distribution of all ratings appears well distributed over the entire range, it is clear that individually *worst*- and *best*-rated HRTFs were consistently at the extremes of the rating scale. This reliability in rating performance agrees with previous studies employing the same methodology^25,28^.

The number of times each of the 7 HRTFs was identified as the *best* or *worst*-match is shown in Table 2. It can be observed that each of the 7 HRTFs was identified as both *best* and *worst* at least once by different members of the test subject pool, providing further support of the subjective orthogonality of the HRTF subset.

**Table 2:** Number of times each of the 7 HRTFs was rated as *best* and *worst* in HRTF selection rating for the 16 subjects using non-individual HRTFs.

LISTEN	Times selected	
HRTF ID	*best*	*worst*
IRC_1008	2	1
IRC_1013	1	3
IRC_1022	1	1
IRC_1031	2	3
IRC_1032	6	2
IRC_1048	3	1
IRC_1053	1	5

The previous related study^23^ employed these judgments to evaluate the effect of HRTF quality on adaptation using either the *best*- or *worst*-rated HRTF set for each subject. After 3 training sessions, subjects with their *best*-rated HRTF showed greater adaptation to the HRTF than those with their *worst*-rated one. In the current study, subjects were only given their *worst*-rated HRTF, assuming this to be the worst case scenario for listeners in any practical application, especially those that do not allow for a choice of HRTF. This procedure controls for the unknown degree of similarity, and subsequent bias, between subjects when using a single “generic”/dummyhead HRTF in subjective evaluations, or the limited spectral modifications possible in the use of pinnae inserts.

While a comparable degree of *quality* cannot be assured between different participants *worst* matches (*i.e*. how much worse is the worst HRTF for each individual), this method is an improvement over alternate methods which are either uncontrolled or limited in the extent of spectral changes.

### Tracking and Binaural Rendering

The experiment used real-time dynamic binaural rendering to present the stimuli to the subjects. The binaural rendering was performed using full-phase HRTF convolution with the LIMSI Spatialization Engine^29^, recently released as a free VST audio plugin under the Anaglyph project^30^. Since the aim is to investigate adaptation to the spectral part of the HRTF, subjects tested with non-individual HRTFs had the ITDs personalized in the rendering, based on a morphological estimation model using the head circumference measure^31^. ILDs, which are broadband averages of these spectral differences, were not modified.

Open circum-aural reference headphones (Sennheiser HD 650) were used. No headphone compensation was included. Any such applied equalization, as proposed by some studies, while having been shown to potentially improve the naturalness of binaural rendering of real stimuli, would not be relevant in the current study where simple noise bursts are used. Such stimuli have no “naturaless”. In addition, such equalization, being applied globally irrespective of virtual source position, acts simply as omni-directional source coloration filters, and therefore should not affect basic localization tasks. Head-tracking, as well as the tracking of a hand-held object given to the subjects, was carried out using an 18 camera OptiTrack system with a latency of 10–15 ms, which is below the general threshold of detectability for VAE^32^.

### Experiment Design and Procedure

Each experimental session consisted of two tasks, an adaptation or *training game* and a *localization test* for accommodation assessment. Both tasks are the same as those used in^23^, which can be consulted for full details.

In order to test if adaptation to non-individual HRTFs is retained for long periods of time, the current study was carried out over the course of several months, with a total of 10 sessions. The first 4 sessions included both the training game and localization test for all subjects. This was to give all subjects the chance to adapt to their individually assigned HRTF set. These first 4 sessions took place once a week for 4 consecutive weeks. After session 4, subjects were split into two groups of 8 subjects such that the group’s polar angle error distributions were equivalent (see Sec. Group divisions for details); subsequent sessions were once every two weeks. Group **W10** continued with the training game until the end of the 10 sessions. Group **W4** carried out only the localization test for sessions 5–10, no longer performing the training game after session 4. Finally, the control group **C10**, with 4 subjects, performed the sessions with the same frequency and did both the training game and localization test for the 10 sessions, as per **W10**. The control group used their own HRTFs which were previously measured as part of the French national research project BiLi^33^. The BiLi HRTF measurement protocol has been compared to that of LISTEN with high compatibility^34^.

The training procedure was devised as a simple game with a searching task in which the listener had to find a target at a hidden position in some direction (*θ*,*ϕ*) in the head-centered space around them. Subjects searched for the hidden target by moving the motion-tracked hand-held object in the space around their head. Subjects started each trial facing forward in the chair with their head in an upright position, and were requested to place the hand-held tracked object on their leg. The trial would not start unless the object was at an elevation angle of ≤−55° relative to their forward facing head. For the duration of the search, alternating pink/white noise (50–20000 Hz) with an overall level of approximately 55 dBA measured at the ear was presented to the listener through the binaural renderer, positioned at the location of the tracked hand-held object relative to the subject’s head. This provided a link between the proprioceptively known position of the subject’s own hand and the spatial cues in the binaural rendering. The alternation *rate* of the pink/white noise bursts increased with increasing angular proximity to the target direction (the radial distance of the hand and hidden target were not considered when searching), using a Geiger counter metaphor^35,36^. Once the subject reached the intended target direction, an animal sound would play, spatialized at the target’s location. After finding a target, the subject returned to the initial position and, to allow for short pauses, pressed the mouse button (wireless mouse on a necklace) to pass to the next trial. The training game lasted 12 min and subjects were instructed to find as many animals as possible in the time available. It should be emphasized that no auditory localization on the part of the subject was actually required to accomplish this task, only tempo judgment of the alternation *rate* of the pink/white noise bursts and proprioceptive knowledge of one’s hand position. HRTF adaptation is therefore a result of game play, but not the task of the game as far as the participant is aware. In contrast to audio-visual feedback target training used in previous studies, this task has been designed to facilitate learning positions outside of the visual field of view, as well as to function for individuals with visual impairments.

The second part of the experiment was the localization test. Again, subjects were asked to start facing forward and with their head straight, and the hand-held tracked object on their knee. As with the training game, the trial would not start unless the object was at an elevation angle of ≤−55° relative to their forward facing head. To facilitate subjects’ orientation, there was a physical marker placed on the floor that they could find with their feet. To evaluate localization, subjects would hear a brief burst of noise (to limit the influence of any possible head movement during playback) and would subsequently point in the perceived direction of the sound using the hand-held object. The noise burst consisted of a train of three, 40 ms Gaussian broadband noise pulses (50–20000 Hz) with 2 ms Hann ramps at onset and offset and 30 ms of silence between each burst^23^. The overall level of the train was approximately 55 dBA measured at the ears. Subjects were seated on a swivel chair to aid in the indication of directions to the rear, as some limitations could occur in pointing tasks to rear locations due to physical constraints^37^. The perceived orientation was calculated between the initial head-center position/orientation when the stimulus was played and the final hand position when the subject validated the target. No feedback was given to subjects regarding the target position. There were 25 target directions with 5 repetitions of each target. The partial sphere included a full 360° of azimuth, and −40° to 90° of elevation relative to ear level. The distribution was the same as that used previously^23^. Subjects were naive to the spatial distribution of the targets. The average duration of the localization test was 10 min.

### Group formations

As stated above, after session 4, subjects assigned non-individual HRTFs were split into two groups of 8 subjects such that each group’s polar angle error distributions were equivalent. Subjects were assigned to groups **W4** and **W10** *post facto*, at the end of session 4, with the intention that group performances at sessions 1 and 4 would be as similar as possible. However, due to scheduling issues, subjects performed the experiment with two different starting dates, with 10 of the 16 starting earlier than the remaining 6 subjects. As such, the first portion of the pool to reach session 4 were split into **W4** and **W10** before the results of the remaining 6 subjects were known. This group assignment was performed by finding the distribution of subjects that gave the highest *p*-values for Kruskal-Wallis tests performed on the total combined localization test response results for both session 1 and session 4.

Once the remaining 6 subjects reached session 4 they were divided between groups **W4** and **W10**, again ensuring the highest possible *p*-values when testing between **W4** and **W10** performance at sessions 1 and 4. It should be noted that, following the assignment of the two groups, one subject from the first 10 subjects dropped out and had to be replaced with a subject starting at a later date. Once all subjects were assigned to their respective groups, following the staggered starting dates and subject replacement, the final *p*-values were 0.15 and 0.75 for sessions 1 and 4 respectively, thus satisfying the intended criterion.

The control group **C10** were selected based solely on the availability of their HRTF data and their availability for the study.

### Spatial coordinates and data analysis

Analysis of localization performance was performed using the interaural polar coordinate system^38^. In this coordinate system, the direction of a vector between the head center and a point on the sphere is expressed by two angles: the lateral angle and the polar angle. The angle between the vector and the median plane is the lateral angle, from −90° to 90°. The polar angle corresponds to rotation around the interaural axis, from −90° to 270°, with 0° being directly in front. This is a natural coordinate system for human localization data since it allows for the rough separation of interaural cues, which are related to the ITD/ILD and are represented by the lateral angle, from the spectral cues, which are related to the HRTF and are represented by the polar angle. Localization errors in lateral and polar angles were analyzed by examining the magnitude of the difference between the target angle and the perceived angle.

**Figure 2:**
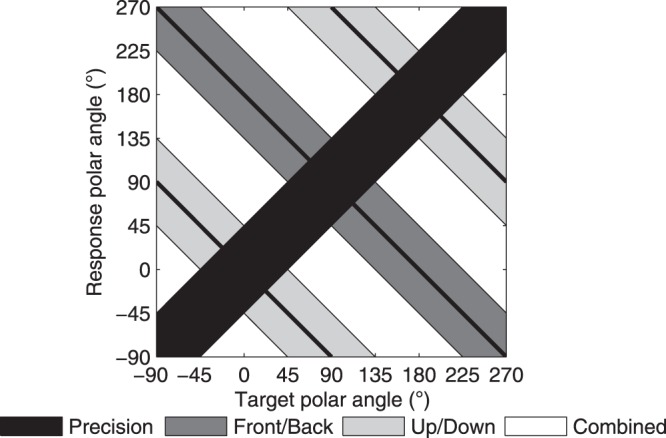
Response classification analysis: Definition of the four different response type classification zones: *precision*, *front-back* error, *up-down* error, and *combined* error (from^23^).

Using the interaural polar coordinate system, all front-back and up-down confusion errors are contained in the polar angle, as defined above. Polar responses can be grouped into four categories as a function of type: *precision*, *front-back* error, *up-down* error, and *combined* error (see^23^ for more detailed definitions). These are summarized in Fig. 2. In short, responses classified under *precision* are for those within ±45° of the target angle, *front-back* classified errors are responses reflected in the frontal plane, and those classified *up-down* are for those reflected in the transverse plane. Any responses that fall outside of these regions are classified as *combined* type errors.

## Results

### Localization test

The localization test collected both lateral and polar angles for the direction indicated by the subject. Spectral adaptation results primarily in improvements in polar angle responses. Consequently, analysis of lateral error is provided separately in Sec. Lateral Angle Error.

#### Polar Angle Error

**Figure 3:**
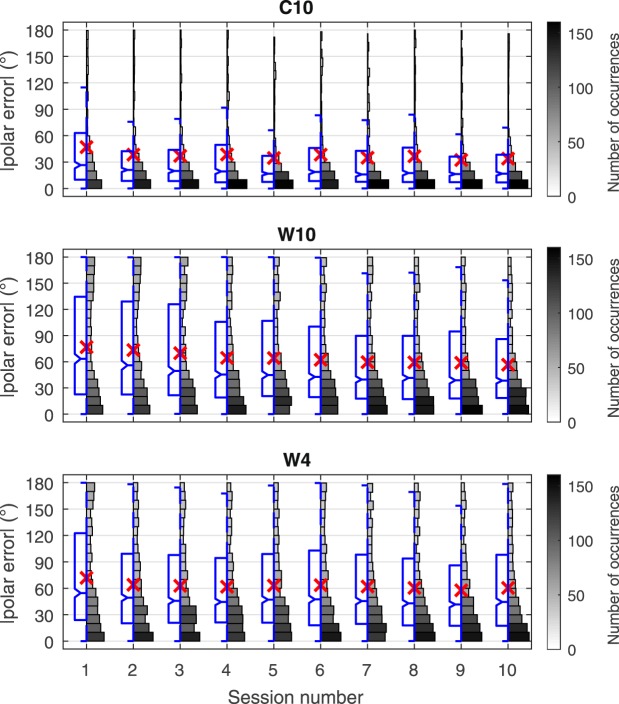
Polar angle error evolution over the 10 sessions for the three participant groups. Split boxplot-histogram of the magnitude of polar error by session, results for all subjects combined by group. Boxplot and angular error axis scales (left); mean values (×); histogram value gray-scale maps provide numerical data references for number of occurrences of responses represented by the histograms (right). Boxplot diagrams indicate the 25^th^ percentile (*Q*1), median (*Q*2, comparison interval notch with significance *α* = 5%), and 75^*th*^ percentile (*Q*3), with data extent whiskers spanning the lowest and highest data points in the range *Q*1 − 1.5 × (*Q*3 − *Q*1) to *Q*3 + 1.5 × (*Q*3 − *Q*1).

Figure 3 shows a summary of the absolute polar error over all subject responses with a representation combining boxplot, mean magnitude error, and histogram for each group as a function of localization test session. This type of representation has the advantage of combining a boxplot (left side) containing traditional statistical data with a histogram (right side), representing the distribution of the response errors. The polar angle contains all front-back and up-down confusions; no resolution or suppression of these types of errors was performed in order to observe their evolution on the distribution of the responses. Due to the nature of polar coordinates, non-precision (*i.e*. confusion) type errors manifest as bi/multi-modal polar error distributions in the histogram representation.

At session 1, there is a significant difference between the control group and the two groups using non-individual worst-rated HRTFs. As expected, the error is significantly lower for the control group, showing the superior performance possible when using individual HRTFs. The control group exhibits a normal distribution in session 1, with a small, though not statistically significant (*p* = 0.104), improvement at session 2. Following the improvement, the error stays approximately constant for the remaining sessions, showing little or no effect of training. The slight improvement from session 1 to session 2 is almost entirely due to the influence of one subject exhibiting 100% front-to-back confusions in session 1 (Sec. Individual Subject Examples subsequently examines individual results in more detail). In subsequent sessions, the number of confusions reduced, lowering the overall error for the group.

The two groups with non-individual HRTFs show a general decrease in polar angle error over the first 4 sessions. In session 1, the distribution is bi-modal, as can be observed in the histograms in Fig. 3, due to a large number of *front-back* type errors. By session 4, the peak near 180° error has decreased significantly, with the error being compressed to lower error values. This resolution from bi-modal to uni-modal can be associated with the resolution of *front-back* type errors. Between sessions 1 and 4, there was a general improvement of 11.9° in the median polar angle error for groups **W4** and **W10** combined.

**Figure 4:**
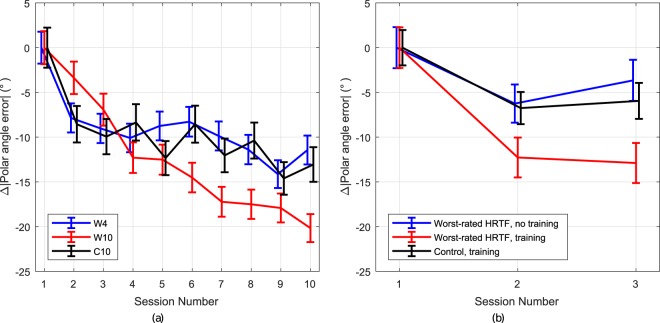
Evolution of mean polar angle error (PAE), calculated across all subjects, normalized in reference to group mean results of session 1, with standard error. (**a**) PAE for the three subject groups **W4**, **W10**, and **C10** over the course of 10 sessions. (**b**) Comparative data: PAE calculated for *worst*-rated HRTF and Control subject groups in Parseihian & Katz^23^ over the course of 3 sessions. No correction has been applied to resolve any quadrant confusions.

To more clearly show the group trends, Fig. 4a shows the group change relative to the mean performance at session 1. From this, group **W10** shows continued reduction of polar angle error over the course of the experiment. Between sessions 4 (when training stopped) and 10, a linear regression analysis across subjects indicates an improvement of −1.34°/session. This is more than twice the rate observed for group **W4**, −0.64°/session, who did not perform any training over sessions 5–10, as well as group **C10**, who exhibited a similar rate of −0.73°/session. The comparable performance improvements observed in group **W4** in the absence of training and group **C10** with individual HRTFs and continued training is unclear, since group **W4** was given no feedback on their performance or the target positions. This point is discussed further in the Discussion section Comparison to Previous Studies below.

#### Individual Subject Examples

**Figure 5:**
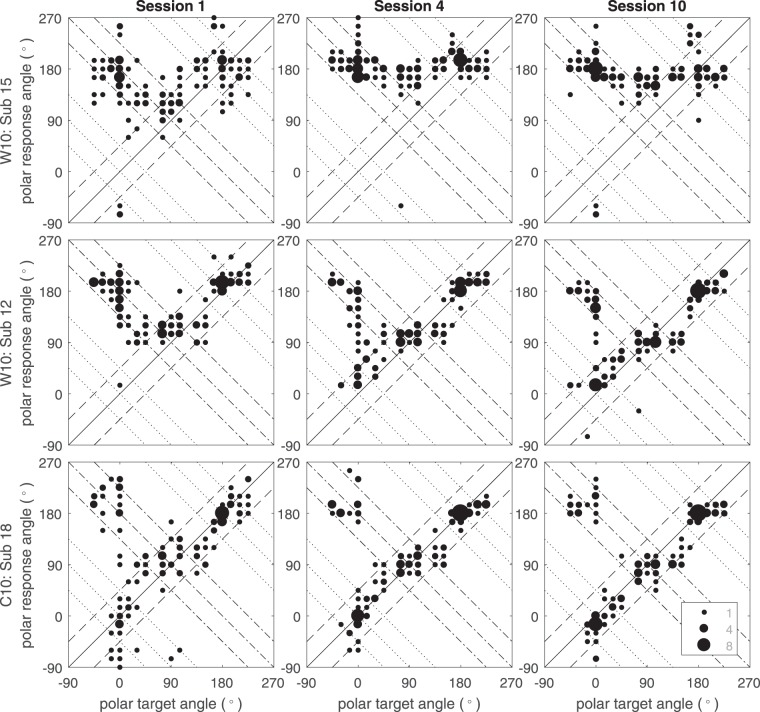
Polar angle responses of three subjects at sessions 1, 4, and 10 for successful (Sub 12) and unsuccessful (Sub 15) accommodation from group **W10**, as well as one of the control subjects (group **C10**).

A large degree of variation in the success of subject adaptation to *worst*-rated HRTF cues was observed. For example, in group **W10** some subjects managed to reach localization accuracy similar to that of the control group by the end of the study. Others did not resolve front-to-back or back-to-front errors, even after the full 10 sessions of training. Figure 5 shows example localization test results for sessions 1, 4, and 10 for subject *Sub 15* who persisted with front-to-back reversals, *Sub 12* who reached near-control group accuracy, and *Sub 18* as a control reference. *Sub 12* and *Sub 15* represent the extremes of adaptation results to the *worst* case HRTFs, with most subjects generally improving to a level somewhere between the two. The control subject shows the same pattern for the duration of the experiment. *Sub 12*’s mean absolute polar error improved from 70.7° in session 1, 49.0° in session 4, to 34.7° in session 10, compared to **C10** group means of 47.0°, 38.7°, and 34.0°. *Sub 15* showed no such improvement and was unable to resolve front-back confusions for the duration of the study.

As well as the absolute polar error, performance is analyzed via response classification. Control group subject *Sub 18*’s results are generally accurate, with most responses falling within the *precision* response classification region, with a small number of *front-back* confusions for sources with zero or negative polar angles. By contrast, *Sub 12* and *Sub 15* exhibited similar performance in session 1, with almost complete front-to-back reversals. By session 4, *Sub 12* improved, reducing the number of *front-back* errors. A similar but less marked training effect can be observed up to session 10, resulting in further resolution of *front-back* type errors. The distribution of response classifications as *precision*, *front-back*, *up-down*, and *combined* for *Sub 12* at session 10 are 78.4%, 6.4%, 4.0%, and 11.2% respectively, compared with mean classification rates of 79.0%, 6.6%, 0.8%, and 13.6% for group **C10**.

In all comparisons, no specific trends were observed with regards to the background or demographics of those subjects who where more or less successful at adapting to their *worst*-ranked HRTF.

#### Subject Grouping By Improvement Rate

As exhibited in the previous section, the amount of improvement with continued training over sessions 5–10 is highly individual. As a measure of accommodation, the *rate of improvement* was defined as the gradient of the linear regression of PAE over sessions 5–10 for individual subjects. The rates of improvement for the 8 subjects in group **W10**, in ascending order, were [0.5, 1.0, 1.2, 1.6, 1.9, 2.4, 3.9, 4.6]°/session over sessions 5–10. For the 4 subjects in group **C10**, the rates of improvement over the same sessions were [0, 0.9, 1.0, 2.2]°/session. Taking into consideration these individual results, for further analysis group **W10** is divided into two subgroups: separating those whose rate of improvement exceeded the maximum observed rate of improvement in the control group **C10** of 2.2°/session (subgroup **W10+**, comprising 3 out of the 8 subjects) and the remaining subjects (**W10**−) who did not exhibit clear HRTF adaptation results based on this criterion over and above that of the control group.

**Figure 6:**
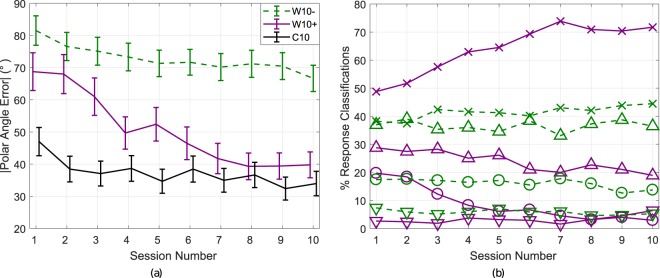
Result analysis by subgroup. (**a**) Mean absolute polar angle error and 95% confidence intervals for groups **W10+**, **W10−** and **C10** across sessions 1–10. (b) Response classification analysis: Mean classification of results for group **W10** by type (*precision* (×), *front-back* error (◯), *up-down* error (∇) and *combined* error (Δ)) for groups **W10 + **(**—**, 3 subjects) and **W10−** (**—**, 5 subjects) over sessions 1–10.

The mean absolute polar error with 95% confidence intervals is shown in Fig. 6a for **W10+**, **W10**−, and **C10**. Group **W10+** approaches a similar level of absolute performance to **C10**. This demonstrates that these subjects were able to adapt well to their *worst*-rated HRTF to a level approaching subjects using their individually measured HRTF. It also shows clearly that despite continuous training some subjects exhibited little or no improvement.

The response classification results for **W10+** and **W10**− are shown in Fig. 6b. At the outset of the study, it can be observed that *up-down* and *front-back* type error rates are comparable between the two subgroups, with **W10**− exhibiting more *combined* type errors. This metric could be a potential indicator for identifying poor HRTF adaptation conditions. Subsequently, it can be clearly seen that **W10+** exhibits a steady increase in *precision* classified responses, with reductions in *front-back* errors over sessions 3–5 and subsequent reductions in *combined* errors. In contrast, **W10**− exhibits generally consistent response classifications across sessions, with only small increases in *precision* classification mirrored by a decreasing trend in *front-back* errors. For all subjects, it can be noted that the occurrence of *up-down* errors is quite rare.

### Lateral Angle Error

**Figure 7:**
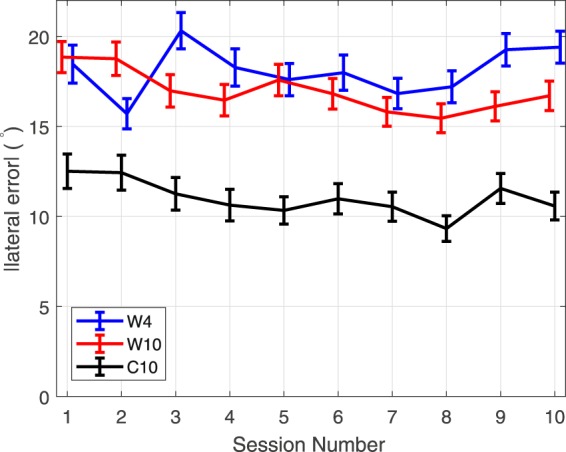
Mean absolute lateral error during the localization tests for groups **W4**, **W10**, and the control group **C10** for each of the 10 sessions. Vertical bars indicate 95% confidence intervals.

The mean absolute lateral error, representing predominantly ITD/ILD cues, for each of the localization tests is shown in Fig. 7 for each group over the course of the 10 sessions. Localization blur ranges from 9.3° to 12.5° for the control group **C10** and 15.5° to 19.4° for groups **W4** and **W10** using non-individual HRTFs. The absolute error for the control group is lower than for the two test groups for all sessions. This is most likely due to the use of individual ITD cues, while the non-individual HRTF groups are using an individualization approximation based on head circumference.

During sessions 1–4 group **C10** shows a slight improvement of approximately 2° reduction in the mean error, with marginal significance (Tukey’s HSD test, *p* = 0.0647). For the two groups using non-individual HRTFs there is no trend in improvement during the first 4 sessions, indicating no strong learning effect for lateral localization.

Taken over the full 10 sessions, the control group shows a trend for reduced error with a borderline statistically significant improvement between sessions 1 and 10 (Tukey’s HSD test, *p* = 0.0499). For group **W10** there is a slight trend to improvement during the 10 sessions, with a trend from linear fit of −0.29°/session. This slow rate of learning indicated by the linear fit suggests that there was no appreciable learning effect. Group **W4** shows no clear pattern of learning over sessions 5–10 as would be expected without the training task, and there is no significant difference between sessions 1 and 10 (Tukey’s HSD test, *p* = 0.9309). Over sessions 5–10, after stopping the training game, there is a slight trend of increasing error. As there was no consistent improvement observed over the first 4 sessions, this suggests that the trend in sessions 5–10 is not related to loss of adaptation but rather response variance.

Overall, the effect of training on the lateral error was slight at best, suggesting there has not been significant adaptation to ITD/ILD cues using this training game. An average across sessions 1–10 gives a general absolute lateral error of 11°, 17°, and 18° for groups **C10**, **W10**, and **W4**. This discrepancy would appear to indicate that the ITD approximation model used does not provide “ideal” personalization to the level of individual HRTFs. This will be addressed in future works through improvements to the model.

## Discussion

### Comparison to previous studies

For comparison to Fig. 4a,b shows the standard polar angle error calculated for the previous rapid training study^23^ for the relavent groups to the current study – those using their worst-rated HRTFs, both with and without training, and an individual HRTF control group. Subjects with their worst-rated HRTF in the current study exhibit similar improvement to those of the previous study where the training game was used, *i.e*. improved by at least 10° after three training sessions. In the current study, similar improvement was obtained after four sessions for groups **W4** and **W10**, although group **W4** did not fully obtain the same improvement. However, average performance between groups **W4** and **W10** gives broadly similar performance to the worst-rated HRTF group with training from the previous study. This demonstrates a similar ability to adapt to non-individual HRTFs, even with the increased time between training sessions in the current study compared to the previous one.

Examining results without training, it can be observed that the group Worst-rated HRTF, no training exhibited similar improvement tendencies as the Control, training group. These previous results reflect the same trends as observed in the current study for groups **W4** and **C10**. It can be noted that the small variations in polar angle error across sessions without training are generally within standard errors for both studies, which may statistically give rise to slight “improvement” rates depending on the precise sessions employed for analysis.

**Figure 8:**
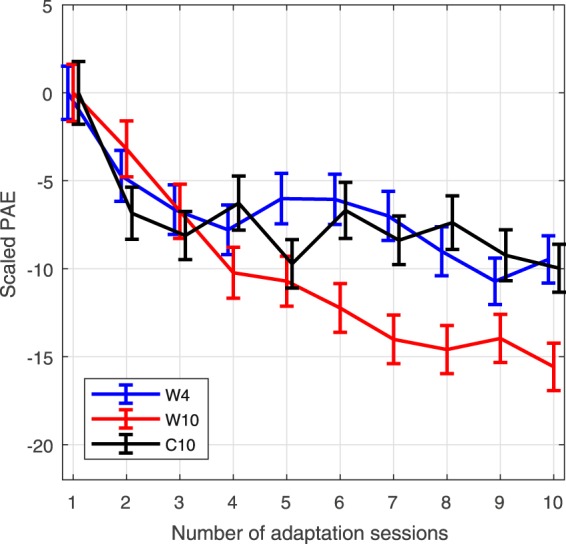
Scaled PAE (mean across all subjects in a given group for each session) with front-back confusions resolved (with standard error) for the three subject groups **W4**, **W10**, and **C10** over the course of 10 sessions, relative to the mean results of session 1.

A study by Carlile *et al*.^12^ previously tested adaptation to spectral cues modified by having subjects wear pinnae insert molds. Over the course of 10 days, ear molds were worn throughout waking hours which included multiple training sessions. To provide a direct comparison of data analysis to said study, Fig. 8 presents the polar angle error with front-back confusions resolved (in contrast to results presented in previous sections) then scaled by the cosine of the target lateral angle (scaled PAE), as per Carlile *et al*.^12^, for the three subject groups relative to the mean PAE of session 1. This metric facilitates observation of relative improvement over time. It should be noted that the method of identifying front-back confusions in this instance, for comparison purposes, follows the method of Carlile *et al*. (contrary to Fig. 2 employed in the previous sections) where a response is classified as a front-back confusion when the target and response angles are in different hemispheres, with an “exclusion” zone of ±15° around the interaural axis.

The largest improvement observed in Carlile *et al*.^12^ was almost 5° in scaled PAE. In the current study, both **W4** and **W10** reached or surpassed this level of improvement after 3 training sessions. Between sessions 4 and 10, group **W10** further reduces scaled-PAE by approximately 5°. This could indicate that the use of pinnae insert molds to deform the individual’s HRTF only produces minor changes relative to a truly non-individual HRTF, leaving less room for improvement. It should also be noted that Carlile *et al*.^12^ tested for targets only within ±40° of the transverse plane, while the current study tested a wider range of target positions. As such, further comparison of results is not necessarily meaningful.

#### Hypothesis 1: Adaptation to worst-rated HRTF

The results of the first 4 sessions show similar trends to a previous study which applied training over three short-interval training sessions^23^. Concerning polar error, the trend for the first 4 weeks is the same between all non-individual HRTF subjects (groups **W4** & **W10**) and group **B3** (worst-rated HRTF, 3 training game sessions) from^23^. This suggests that the training was similarly effective, even though there was a gap of 1 week between each of the first 4 sessions, while in the previous study all training sessions were contained within a single week, supporting hypothesis **H1**. It appears that training can be similarly effective even when the training game sessions are not carried out in quick succession. In addition, this result already hints at a good retention of the adaptation for at least one week.

Regarding training intervals, it is not immediately clear if the lower rate of improvement for group **W10** during sessions 5–10, compared to sessions 1–4, is due to a saturation effect or the increase in time between the training game sessions. Previous studies^16^ suggest an exponential trend for HRTF adaptation, which would match well the observed trend for **W10**. If learning is retained almost fully between sessions, as the results of several subjects in group **W10** suggest, then the diminished returns must be due to the inability of subjects to further adapt to their worst case HRTF set.

#### Hypothesis 2: Continued adaptation over extended training duration

By session 10, group **W10** had reached a similar level of polar angle error, as shown in Fig. 3, to the group with their *best*-rated HRTF set (**G3**) after 2 sessions in Parseihian & Katz^23^ [see Fig. 2]. The mean improvement of the control group **C10** was 10° between sessions 1 and 10. Over the same period, group **W10** improved by 25°, suggesting 15° can be attributed to adaptation to the HRTFs. This is similar to the improvement observed in the previous study with 3 training sessions, perhaps indicating that the increased time between the training game sessions reduced their effectiveness. It might be that the time interval between training games has an influence, as well as the number, although further investigation would be required to fully determine the effect of this.

Observed polar angle error (and scaled PAE error) rates showed continued improvement by group **W10** when compared to the control group **C10**, who performed the same tasks but using individual HRTFs. The control group exhibited a plateau effect on improvement after just a few sessions. This marked difference in improvement clearly supports hypothesis **H2**.

#### Hypothesis 3: Perennity of adaptation after training period

Mendonça *et al*.^22^ found that localization performance was retained after 1 month without training using the same dummyhead HRTF for all subjects. Zahorik *et al*.^21^, also using a “generic” HRTF for all subjects, found that front-back confusions remained reduced 4 months after training. Using ear inserts molds, Trapeau *et al*.^20^ found that adaptation was retained for at least 1 month, though with decreasing efficiency. Their training task was a combination of listeners wearing ear inserts for 6 days and an active training task that required subjects to turn to face a sound. In the current study, subjects who stopped the training game retained their session 4 polar error performances for the remaining 3 months of the experiment. The control group of ^22^, who were retested 1 month after training (and did not perform intermediate evaluation), showed reduced retention of performance, suggesting at least some possibility of procedural influence. Trapeau *et al*.^20^ also considered procedural effects to explain at least part of the observed loss of HRTF learning upon retesting. In the current study, the evaluation localization test was performed every 2 weeks after the first 4 sessions, and no general degradation of performance was found.

The results of group **W4**, when compared to group **W10**, indicate that polar errors generally remained stable, or slightly improved, in the absence of the adaptation game task. Any observed improvement in localization error by **W4** after session 4 can only reasonably be associated to a learning/attention effect of the localization test, as it must be emphasized that there was no feedback provided in this task.

Regarding accommodation in the absence of feedback, a previous study by Mendonça *et al*.^14^ did not observe any improvement in localization with naive subjects using a non-individual HRTF over the course of 10 sessions. That study employed *octant* source position reporting restricted to the horizontal plane and reflects the observed performance in the current study concerning the lack of improvement in lateral angle error across sessions and groups. In contrast, inspection of the results of Zahorik *et al*. in a study which examined in fine detail the moment of improvement in localization with training showed a slight tendency in reduction of errors during the post-training phase^39^, [see Fig. 5]. While that study used only a few subjects, such results are comparable to those observed in the current study, where albeit small improvements were observed after the cession of training, and such improvements were far less than the improvements observed while training continued. Therefore, the slight improvement observed for group **W4** may indicate that subjects had become more attentive and aware of spectral localization cues due to continued testing. While a linear regression analysis of polar angle errors showed comparable slight improvements over later sessions by both groups **W4** and **C10**, comparing across sessions shows that these fluctuations are within standard error in almost all cases, in contrast to results for **W10** which show clear improvement.

As such, following these, it could be reasonable to hypothesize that continued exposure to a given HRTF, comprising a wide range of source positions, allows the auditory system to construct a possibility map based on previous events, regardless of feedback. For example, becoming able to discern front/back confusions or to refine cone-of-confusion solutions by building on ITD/ILD/inter-spectral differences and adapting those to the new HRTF over time. This type of unsupervised learning may be comparable to passive HRTF adaption studies where no specific training task was performed. However, the very minor degree of improvement by **W4** after active training stopped does not lead one to believe that, if present, this would be an effective means of training. As groups **W4** and **C10** showed similar performance, and since **C10** performed training which **W4** did not, the stability of observed polar angle errors by **W4** after training stopped supports the retention of HRTF learning over time, confirming hypothesis **H3**. However, due to the potential impact of continued testing (in the absence of feedback), attention must be given to these results and their implication in past and future studies regarding the impact of localization tests alone on accommodation to non-individual HRTFs.

#### Hypothesis 4: Generality of adaptation to worst-rated HRTF

Previous studies investigating spectral adaptation using ear molds have required subjects to wear them continuously for days at a time. By comparison, the 12 minute training sessions in this study were very short. In spite of this, the scaled-PAE error improvements over the first four sessions, shown in Fig. 8, are approximately 7° or greater for groups **W4** and **W10**. This is a greater improvement than exhibited by subjects in Carlile *et al*.^12^ who wore pinnae insert molds continuously for 10 days and performed several training tasks. The reasons for the differing magnitudes might be caused by the degree of spectral dissimilarity when using worst-rated HRTFs or ear molds. Simply, the spectral difference between a worst-rated HRTF and a subject’s own HRTF is potentially greater than when using ear molds. Such a condition would allow for a greater range of improvement since adaptation is beginning from a point further from the ideal. It might also be that different localization test methodologies, such as different target distributions, explain some of the difference in reported results.

However, in the current study the observed adaptation ability of subjects was found to span a spectrum from those exhibiting near complete adaptation to an apparent inability to adapt to their worst-rated HRTF. Diminishing returns on the benefit of training was observed, possibly as a consequence of the selection of the perceptually worst performing HRTF. It is possible that the training task does not work for some subjects when they are given such degraded localization cues. Alternatively, HRTF mismatch could be multidimensional, with some differences being able to be addressed through training, and others not. Parseihian & Katz^23^ showed that appropriate selection of the *best*-matched HRTF allowed for more rapid and effective adaptation for localization. Without the appropriate choice, it appears that the benefit of training is not guaranteed (it is subject dependent) and that adaptation is slower. Trapeau *et al*.^20^ found similar results for listeners using ear inserts. In analyzing potential indicators for poor adaption results, they reported that adaptability was not correlated to localization performance when tested after initial insertion of the ear molds. Despite the difference between training tasks of the two studies, both results indicate that some subjects might have severe difficulties with, or be incapable of, adaptation with such degraded auditory cues. As a result, hypothesis **H4** is clearly refuted, supporting the notion that adaptation to a worst-match non-individual HRTF cannot be assured for every individual.

## Conclusion

Results of this perceptual study show that adaptation to an individual’s perceptually worst-rated HRTF can continue as long as training is provided, though the rate of improvement decreases after a certain amount of training sessions, and when extended times are allowed between training sessions. A subgroup of the three subjects out of eight improved at a rate faster than the control group over sessions 5–10, achieving localization performance levels approaching the control group with individual HRTFs. These performance levels are comparable to those observed in a previous study with identical test protocol, where subjects performed only 3 training sessions using their perceptually best-rated HRTF.

With regards to the perennity of HRTF adaptation, results showed relative stability in localization performance after training stopped. Due to the observation of a slight trend in performance after training ceased, it is difficult to *entirely* separate retention of HRTF adaptation and testing procedure related improvements for polar error measures over the course of 3 months. Such learning leads to the hypothesis that initially naive subjects became more aware of localization cues during the course of the study, improving in the task accordingly in the absence of feedback. Such results raise concerns about the outcomes of extended studies using initially naive subjects. Future studies should consider localization task repetition training to ensure stable performance prior to any HRTF modification procedures.

Performance improvements in localization through accommodation were found to be highly variable, with some subjects reaching levels comparable to those with individual HRTFs while others failed to exhibit any significant improvement (*i.e*. continuing to show large numbers of front-to-back confusions). Adaptation to perceptually poorly-rated HRTFs has already been found to be slower than for well-rated ones^23^.

The overall implication of these results highlight the need for VAEs to include some form of HRTF selection, as successful adaptation to poorly matched HRTFs appears limited. Using a single generic HRTF for all users will lead to the likelihood that a proportion of listeners will be using an HRTF that is far from their perceptual best, approaching their perceptual worst. Within this group, adaptation will be slower and some listeners may not be able to adapt to the cues which greatly vary with respect to their own individual HRTF, resulting in a poor user experience. If a single HRTF is to be used for all subjects, some form of perceptual rating should be performed before any training in order to better interpret the results and contextualize any variability in adaptation rates. This would help differentiate between slow adaptation as either something inherent in the subject or being due to the perceptual proximity of the tested HRTF. Parseihian & Katz^23^ showed subjects with a well-rated HRTF reached near control levels of performance for polar angle error after 3 training sessions. This suggests the proportion of subjects unable to adapt is likely to be much lower when HRTFs are perceptually better rated.

## Data Availability

The datasets obtained and analyzed during the current study are available from the corresponding author on reasonable request.
